# Transcranial Doppler combined with quantitative EEG brain function monitoring and outcome prediction in patients with severe acute intracerebral hemorrhage

**DOI:** 10.1186/s13054-018-1951-y

**Published:** 2018-02-20

**Authors:** Ying Chen, Weihai Xu, Lijuan Wang, Xiaoming Yin, Jie Cao, Fang Deng, Yingqi Xing, Jiachun Feng

**Affiliations:** 1 Department of Neurology, The First Norman Bethune Hospital of Jilin University, Changchun, Jilin 130021 China; 2 Department of Neurology, Peking Union Medical College Hospital, Chinese Academy of Medical Sciences and Peking Union Medical College, Shuaifuyuan 1, Dong Cheng District, Beijing, 100730 China

**Keywords:** Transcranial Doppler, Quantitative electroencephalography, Intracerebral hemorrhage, Brain function monitoring, Prognosis

## Abstract

**Background:**

Neurological deterioration after intracerebral hemorrhage (ICH) is thought to be closely related to increased intracranial pressure (ICP), decreased cerebral blood flow (CBF), and brain metabolism. Transcranial Doppler (TCD) is increasingly used as an indirect measure of ICP, and quantitative EEG (QEEG) can reflect the coupling of CBF and metabolism. We aimed to combine TCD and QEEG to comprehensively assess brain function after ICH and provide prognostic diagnosis.

**Methods:**

We prospectively enrolled patients with severe acute supratentorial (SAS)-ICH from June 2015 to December 2016. Mortality was assessed at 90-day follow-up. We collected demographic data, serological data, and clinical factors, and performed neurophysiological tests at study entry. Quantitative brain function monitoring was performed using a TCD-QEEG recording system at the patient’s bedside (NSD-8100; Delica, China). Univariate and multivariable analyses and receiver operating characteristic (ROC) curves were employed to assess the relationships between variables and outcome.

**Results:**

Forty-seven patients (67.3 ± 12.6 years; 23 men) were studied. Mortality at 90 days was 55.3%. Statistical results showed there were no significant differences in brain symmetry index between survivors and nonsurvivors, nor between patients and controls (all *p* > 0.05). Only TCD indicators of the pulsatility index from unaffected hemispheres (UPI) (OR 2.373, CI 1.299–4.335, *p* = 0.005) and QEEG indicators of the delta/alpha ratio (DAR) (OR 5.306, CI 1.533–18.360, *p* = 0.008) were independent predictors for clinical outcome. The area under the ROC curve after the combination of UPI and DAR was 0.949, which showed better predictive accuracy compared to individual variables.

**Conclusions:**

In patients with SAS-ICH, multimodal neuromonitoring with TCD combined with QEEG indicated that brain damage caused diffuse changes, and the predictive accuracy after combined use of TCD-QEEG was statistically superior in performance to any single variable, whether clinical or neurophysiological.

## Background

Spontaneous intracerebral hemorrhage (ICH), especially in patients with severe coma, has a high mortality and disability rate. The pathophysiology of ICH is incompletely understood; there is general agreement that the acute formation of a parenchymal hematoma produces tissue disruption and displacement [1]. Mortality in the early stage after ICH is attributable to increased intracranial pressure (ICP) and tissue shifts [2, 3]. Transcranial Doppler (TCD) is utilized as an indirect measure of ICP because higher ICP causes characteristic changes of decreased end-diastolic flow velocity (Vd) and increased pulsatility index (PI) in the Doppler waveform [4]. Several studies have confirmed that the PI of the unaffected hemisphere may be a predictor of death in acute ICH, suggesting that intracranial hypertension is the most likely cause of death in patients with ICH [5–7]. However, compared to patients with supratentorial cerebral infarction, patients with supratentorial cerebral hemorrhage are more prone to disturbance of consciousness; that is, changes in consciousness state after ICH, which are not always easily explained on the basis of increased ICP, mass effect, or herniation [8]. Relief of elevated ICP by ventricular drainage is commonly performed, and may improve the level of consciousness of patients with ICH with coma. Notwithstanding, coma often persists, implicating other causative pathology [9].

The secondary pathophysiological processes of ICH include progress in cerebral edema [10], activation of apoptotic processes [11], and the toxic effects of hematoma components [12, 13]; these factors can cause severe, persistent damage. Functional imaging with PET indicated that surrounding the hematoma was a region of reduced cerebral blood flow (CBF), cerebral metabolic rate for oxygen (CMRO_2_), and oxygen extraction fraction (OEF) [14]. Combined application of Xenon CT and PET in previous studies on CBF and CMRO_2_ has also shown that EEG changes can reflect the coupling of CBF and brain metabolism [15]. In comparison with primitive EEG, quantitative EEG (QEEG) has an advantage in quantification and interpretation. Delta power measures had the strongest negative correlation with CBF, and alpha power had a relatively strong positive correlation with CBF; increased power in slower frequency bands (delta and theta) and decreased power in faster frequency bands (alpha and beta) are seen with reductions in CMRO_2_ [15–17].

In this study, we sought to investigate whether it is possible to comprehensively evaluate brain function by administering TCD combined with QEEG in patients with severe acute supratentorial (SAS)-ICH, and to assess outcome at the 90-day follow-up; and to explore a new basis for pathophysiological changes in severe ICH.

## Methods

### Patients

In this prospective study, we consecutively enrolled patients with ICH with coma who were admitted to the Department of Neurology, First Hospital of Jilin University, China, between June 2015 and December 2016. The patients were included in our study if they met the following criteria: admission time ≤ 72 h after onset; presence of supratentorial hemorrhage; and Glasgow Coma Scale (GCS) score ≤ 8 points on admission, as assessed by an experienced neurologist. Exclusion criteria were the following: ICH secondary to aneurysm, vascular malformation, tumor, and cerebral infarction; deficient temporal acoustic bone window; scheduled surgical treatments, including ventricular drainage, clot removal, and craniotomies; middle cerebral artery or other intracranial and extracranial major vascular stenosis/occlusion; previous ischemic or hemorrhagic cerebrovascular disease; pathological changes that affect intracranial EEG activity, such as intracranial infection, ischemia anoxic encephalopathy, cerebral trauma, and others; presence of marked environmental turbulence, such as low temperature (core body temperature < 32 °C), hypoglycemia (<50 mg/dl), hyponatremia (<116 mg/dl), and others; and central nervous system depressant use, including sedative, narcotic, antidepressants, antipsychotics, antiepileptic drugs, and others. Fifteen age and sex-matched healthy controls (64.3 ± 13.5 years old, eight men) were recruited. Written informed consent was obtained from each patient’s immediate family members before the beginning of the study. The study was approved by the Ethics Committee of the First Hospital of Jilin University, China, and conformed to the tenets of the Declaration of Helsinki.

### Clinical data

All patients received routine monitoring of vital signs and intensive nursing care in the Neurological Intensive Care Unit. We recorded and analyzed the following variables: demographics (age and sex); stroke risk factors (hypertension, diabetes mellitus, dyslipidemia, atrial fibrillation, previous acute coronary event, smoking, excessive drinking); admission GCS score; time from ICH onset until the time monitoring commenced; whether blood pressure and glucose management were in accordance with current ICH management guidelines [18]; other admission laboratory tests (serum potassium, calcium, and sodium, white blood cell count, platelet count, activated partial thromboplastin time (APTT), International Normalized Ratio (INR)); neuroimaging variables (for regular hematoma, ICH volume was measured using length × width × depth/2; for irregular hematoma, length × width × depth/3 was adopted [19]—head CT with hematoma location and volume on admission was evaluated by a neuroradiologist blinded to the clinical and brain function data); and clinical outcome assessed using the 5-point Glasgow Outcome Scale score 90 days after ictus.

### TCD-QEEG measurements

Patients were in the supine position during quantitative brain function monitoring. TCD was performed using 2-MHz pulsed-wave Doppler probes fixed to each temporal window with a helmet. The depth of acquiring optimal middle cerebral artery signals was 50–60 mm from both sides. Simultaneous EEG was obtained with standard 16-channel electroencephalography with silver chloride scalp electrodes placed in accordance with the 10–20 system; electrode impedance was maintained below 10 KΩ. The healthy control group remained with their eyes closed and awake during the process. Data were recorded for over 30 min until a stable recording was established; the recorded data were stored for further analysis.

### Data analysis

Blinded analysis was performed for each patient. All clearly readable TCD waveforms were used in the calculations. The following variables were analyzed: systolic flow velocity (Vs), diastolic flow velocity (Vd), mean velocity (Vm), and PI from the affected and unaffected hemispheres. Vm was calculated using the following equation:

$$ \mathrm{Vm}=\left(\mathrm{Vs}\hbox{--} \mathrm{Vd}\right)/3+\mathrm{Vd}, $$

and PI was calculated using the following equation:

$$ \mathrm{PI}=\left(\mathrm{Vs}\hbox{--} \mathrm{Vd}\right)/\mathrm{Vm}. $$

Offline QEEG analysis was performed using MATLAB (MathWorks, Natick, MA, USA). After data filtering (high pass 0.3 Hz, low pass 30 Hz) and all segments of artifact-free EEG were analyzed, spectral power was calculated using Fast Fourier transform (FFT) for each electrode over the 1–30 Hz range. The relative power of the delta (1–3 Hz, RDP), theta (4–7 Hz, RTP), alpha (8–13 Hz, RAP), and beta (14–30 Hz, RBP) frequency bands over all channels were used to calculate the global delta/alpha ratio (DAR) and the (delta + theta)/(alpha + beta) ratio (DTABR). The brain symmetry index (BSI) was calculated according to the following formula:

$$ \mathrm{BSI}\left(\mathrm{t}\right)=\frac{1}{M}\frac{1}{N}\sum \limits_{j=1}^M\left\Vert \sum \limits_{i=1}^N\frac{Rij(t)- Lij(t)}{Rij(t)+ Lij(t)}\right\Vert $$

with the power of the signal obtained from a particular hemispheric bipolar channel pair *i* (with *i* = 1, 2, …, *N*) at frequency *j* (or Fourier coefficient, with index *j* = 1, 2, …, *M*), *N* being the number of channel pairs, *M* being the number of Fourier coefficients, *Rij*(*t*) and *Lij*(*t*) for the right and left hemisphere, respectively [20].

### Statistical analysis

All statistical analyses were performed with SPSS version 17.0 (SPSS Inc., Chicago, IL, USA) and MedCalc version 11.4.4 (MedCalc Software, Mariakerke, Belgium). In the univariate analysis, data were reported as mean and SD for normally distributed variables and as median and interquartile range (IQR) for nonnormally distributed variables. Categorical variables were presented as percentages. Student’s *t* tests and median two-sample tests were used for normally distributed variables. Nonparametric Wilcoxon (Kruskal–Wallis) analysis of variance was used for nonnormally distributed variables. The comparison of categorical variables was performed with the chi-squared test. From these analyses, we could select the variables with some prognostic significance (*p* ≤ 0.001). Then, we performed a backward stepwise logistic regression analysis with death at 90 days as the dependent variable. If a TCD or QEEG measure was statistically associated with survival, we calculated its separated and united sensitivity, specificity, and area under the curve with the aid of receiver operator characteristic (ROC) curves. ROC curves were compared by applying DeLong’s test. Calculated two-tailed *p* < 0.05 was considered statistically significant.

## Results

### Basic data

During a 1.5-year period, 76 consecutive patients were diagnosed with severe, acute, spontaneous supratentorial ICH. The following patients were excluded: admission beyond 72 h from onset of symptoms (*n* = 4), surgical procedure (*n* = 2), macrovascular stenosis (*n* = 7), previous cerebrovascular disease (*n* = 5), deficient temporal window (*n* = 6), signal artifacts (*n* = 3), and loss to follow-up (*n* = 2). Finally, we enrolled 47 patients, of which 26 (55.3%) died during the 90-day follow-up period. The median age was 67.3 ± 12.6 years, and 23 (48.9%) of the patients were male. The first TCD-QEEG was performed a mean of 31.0 (19.0–59.0) h after the onset of symptoms. No statistically significant differences between survivors and nonsurvivors were noted for clinical baseline data, including age, sex, risk factors, blood pressure, serum glucose, serum potassium, calcium, and sodium, white blood cell count, platelet count, APTT, INR, and hematoma location. Only larger hematoma volume (*p* < 0.0001) and higher Glasgow Coma Scale score (*p* = 0.001) were associated with mortality (Table 1).

**Table 1:** Demographic and baseline characteristics

Characteristic	All patients (*n* = 47)	Survivors (*n* = 21)	Nonsurvivors (*n* = 26)	*p* value
Demographics				
Age (years), mean (SD)	67.3 (12.6)	68.8 (12.5)	64.1 (12.4)	0.21
Male, *n* (%)	23 (48.9)	9 (42.9)	14 (53.8)	0.45
Risk factors, *n* (%)				
Hypertension	41 (87.2)	18 (85.7)	23 (88.5)	0.77
Diabetes mellitus	7 (14.9)	3 (14.3)	4 (15.4)	0.91
Hyperlipidemia	13 (27.7)	8 (38.1)	5 (19.2)	0.15
Coronary heart disease	13 (27.7)	6 (28.6)	7 (26.9)	0.90
Smoking	15 (31.9)	5 (23.8)	10 (38.5)	0.28
Excessive drinking	11 (23.4)	4 (19.0)	7 (26.9)	0.53
Time from ICH onset to monitor (h), median (IQR)	31.0 (19.0–59.0)	39.0 (19.0–65.0)	26.0 (18.8–46)	0.21
GCS score, median (IQR)	7 (6–8)	7 (7–8)	6 (4–7)	0.001
SBP (mmHg), mean (SD)	168.4 (30.0)	175.3 (25.9)	162.7 (32.4)	0.16
DBP (mmHg), mean (SD)	85.2 (21.3)	90.5 (18.1)	80.9 (23.0)	0.13
WBC (×10^9^/L), mean (SD)	13.0 (3.5)	12.5 (4.2)	13.3 (2.8)	0.44
Platelet (×10^9^/L), mean (SD)	198.3 (78.9)	205.4 (74.5)	192.5 (83.2)	0.58
APTT (s), mean (SD)	29.1 (3.4)	29.1 (3.7)	29.0 (3.1)	0.96
INR, mean (SD)	1.01 (0.09)	0.98 (0.09)	1.01 (0.10)	0.28
Glucose (mmol/L), median (IQR)	7.6 (6.9–9.4)	7.2 (6.8–9.6)	7.7 (6.7–9.2)	0.86
Potassium (mmol/L), mean (SD)	3.6 (0.4)	3.6 (0.4)	3.7 (0.4)	0.33
Calcium (mmol/L), mean (SD)	141.3 (5.5)	141.6 (6.2)	141.0 (4.9)	0.74
Sodium (mmol/L), median (IQR)	2.2 (2.1–2.3)	2.2 (2.1–2.3)	2.2 (2.1–2.3)	0.40
Hematoma side, left, *n* (%)	30 (63.9)	14 (53.8)	16 (76.2)	0.11
Hematoma location, *n* (%)				0.65
Lobe	8 (17.0)	3 (14.3)	5 (19.2)	
Deep	39 (83.0)	18 (85.7)	21 (80.8)	
Hematoma volume (cm^3^), median (IQR)	45.5 (25.0–75.9)	25.0 (19.6–39.6)	62.8 (44.1–90.1)	<0.0001
Intraventricular hemorrhage, *n* (%)	37 (78.7)	22 (84.6)	15 (71.4)	0.27

*SD* standard deviation, *ICH* intracerebral hemorrhage, *IQR* interquartile range, *GCS* Glasgow Coma Scale, *SBP* systolic blood pressure, *DBP* diastolic blood pressure, *WBC* white blood cell count, *APTT* activated partial thromboplastin time, *INR* International Normalized Ratio

### Evaluation of brain function with TCD-QEEG

**Fig. 1:**
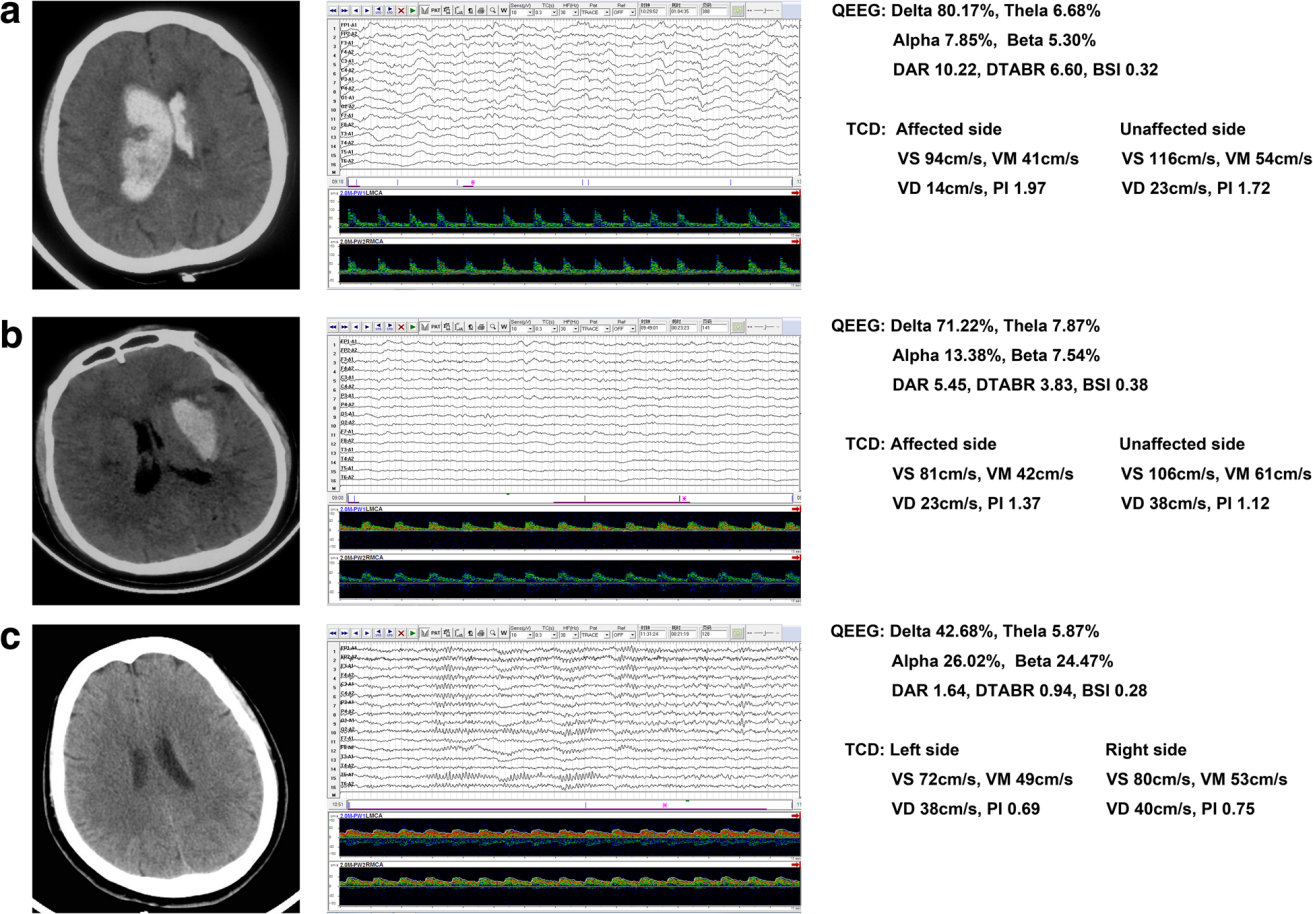
Examples of representative patients. **a** Nonsurvivor patient. QEEG shows the slower delta frequency band significantly increased and the faster alpha frequency band significantly decreased. Moreover, DAR, but not BSI, also increased. TCD shows the PI of bilateral hemispheres significantly increased. **b** Survivor patient. QEEG and TCD show similar changes, but not as significant, to those seen in (**a**). BSI did not increase either. **c** Healthy control patient. QEEG and TCD normal. DAR delta/alpha ratio, DTABR (delta + theta)/(alpha + beta) ratio, BSI brain symmetry index, VS systolic flow velocity, VM mean flow velocity, VD diastolic flow velocity, PI pulsatility index, TCD transcranial Doppler, QEEG quantitative electroencephalography

Figure 1 shows the CT and TCD-QEEG findings of representative patients.

**Fig. 2:**
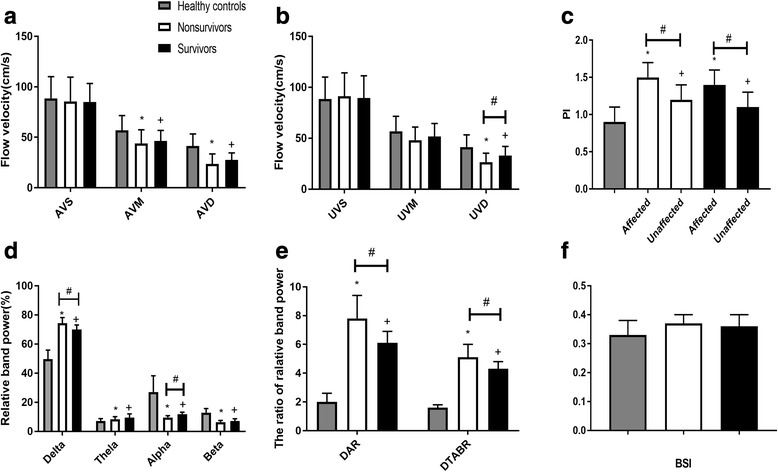
TCD and QEEG parameters in patients with ICH compared to healthy controls. TCD parameters: **a** affected hemisphere systolic flow velocity (AVS), mean flow velocity (AVM), and diastolic flow velocity (AVD); **b** unaffected hemisphere systolic flow velocity (UVS), mean flow velocity (UVM), and diastolic flow velocity (UVD); and **c** pulsatility index (PI). QEEG parameters: **d** relative band power of delta, theta, alpha, and beta; **e** delta/alpha ratio (DAR) and (delta + theta)/(alpha + beta) ratio (DTABR); and **f** brain symmetry index (BSI). ^#^*p* < 0.05 for nonsurvivors vs survivors; *p < 0.05 for nonsurvivors vs healthy controls; ^+^*p* < 0.05 for survivors vs healthy controls

Regarding TCD relevant indicators, Vd of unaffected hemispheres was lower in nonsurvivors (UVd *p* = 0.018) and the PI of both hemispheres was higher in nonsurvivors than survivors in patients with SAS-ICH (API *p* < 0.0001, UPI *p* < 0.0001) (Table 2 and Fig. 2b, c). Vm in the affected hemisphere and Vd and PI in both hemispheres showed significant differences (all *p* < 0.0001) between patients with ICH and healthy controls (Table 2 and Fig. 2a–c).

**Table 2:** TCD and QEEG parameters

	Nonsurvivors (*n* = 26)	Survivors (*n* = 21)		Healthy controls (*n* = 15)
TCD parameters				
VS (cm/s), mean (SD)				
Affected side	85.6 (23.9)	84.9 (18.4)	Overall	88.6 (21.4)
Unaffected side	91.2 (23.0)	89.5 (21.8)		
VM (cm/s), mean (SD)				
Affected side	43.8 (13.6)^*^	46.3 (10.5)^+^	Overall	56.8 (14.8)
Unaffected side	48.0 (12.9)	51.7 (12.7)		
VD (cm/s), mean (SD)				
Affected side	23.6 (9.9)^*^	27.6 (7.0)^+^	Overall	41.3 (12.0)
Unaffected side	26.6 (8.7)^# *^	33.0 (8.9)^+^		
PI, mean (SD)				
Affected side	1.5 (0.2)^# *^	1.2 (0.2)^+^	Overall	0.9 (0.2)
Unaffected side	1.4 (0.2)^# *^	1.1 (0.2)^+^		
QEEG parameters				
RDP (%), median (IQR)	74.4 (72.5–78.2)^#*^	70.0 (67.7–73.1)^+^		49.7 (40.0–55.9)
RTP (%), median (IQR)	8.4 (7.2–10.1)^*^	9.5 (8.2–12.0)^+^		7.1 (6.4–8.8)
RAP (%), median (IQR)	9.5 (8.4–10.8)^#*^	11.9 (10.2–13.2)^+^		27.0 (24.8–38.2)
RBP (%), median (IQR)	6.4 (5.0–7.5)^*^	7.1 (4.2–8.7)^+^		12.9 (9.3–15.7)
DAR, median (IQR)	7.8 (6.8–9.4)^#*^	6.1 (5.4–6.9)^+^		2.0 (1.2–2.6)
DTABR, median (IQR)	5.1 (4.6–6.0)^#*^	4.3 (3.7–4.8)^+^		1.6 (0.9–1.8)
BSI, median (IQR)	0.38 (0.33–0.40)^*^	0.36 (0.31–0.40)^+^		0.33 (0.29–0.38)

*TCD* Transcranial Doppler, QEEG quantitative electroencephalography, *VS* systolic flow velocity, *SD* standard deviation, *VM* mean flow velocity, *VD* diastolic flow velocity, *PI* pulsatility index, *RDP* relative delta power, *IQR* interquartile range, *RTP* relative theta power, *RAP* relative alpha power, *RBP* relative beta power, *DAR* delta/alpha ratio, *DTABR* delta + theta)/(alpha + beta) ratio, *BSI* brain symmetry index

^#^*p* < 0.05 for nonsurvivors vs survivors

^*^p < 0.05 for nonsurvivors vs healthy controls

^+^*p* < 0.05 for survivors vs healthy controls

Regarding QEEG relevant indicators, higher relative delta power (RDP *p* < 0.0001), lower relative alpha power (RAP *p* < 0.0001), higher delta/alpha ratio (DAR *p* < 0.0001), and higher (delta + theta)/(alpha + beta) ratio (DTABR *p* = 0.002) were associated with mortality in patients with SAS-ICH. All indicators except BSI showed significant differences (all *p* < 0.0001) between patients with ICH and healthy controls (Table 2 and Fig. 2d, e). There were no significant differences in BSI between survivors and nonsurvivors or between patients and controls (all *p* > 0.05) (Table 2 and Fig. 2f).

### Multivariate analysis

All *p* ≤ 0.001 variables in the univariate analysis were entered into a logistic regression model with mortality at 90 days as the dependent variable. The result showed that only DAR (OR 5.306, CI 1.533–18.360, *p* = 0.008) and UPI (adjusted OR 2.373, CI 1.299–4.335, *p* = 0.005) were identified as independent predictors for mortality at 90 days after SAS-ICH. The results of the logistic regression analysis remained unchanged when we excluded the GCS score and hematoma volume to verify model stability.

### Comparison of ROC curves

**Fig. 3:**
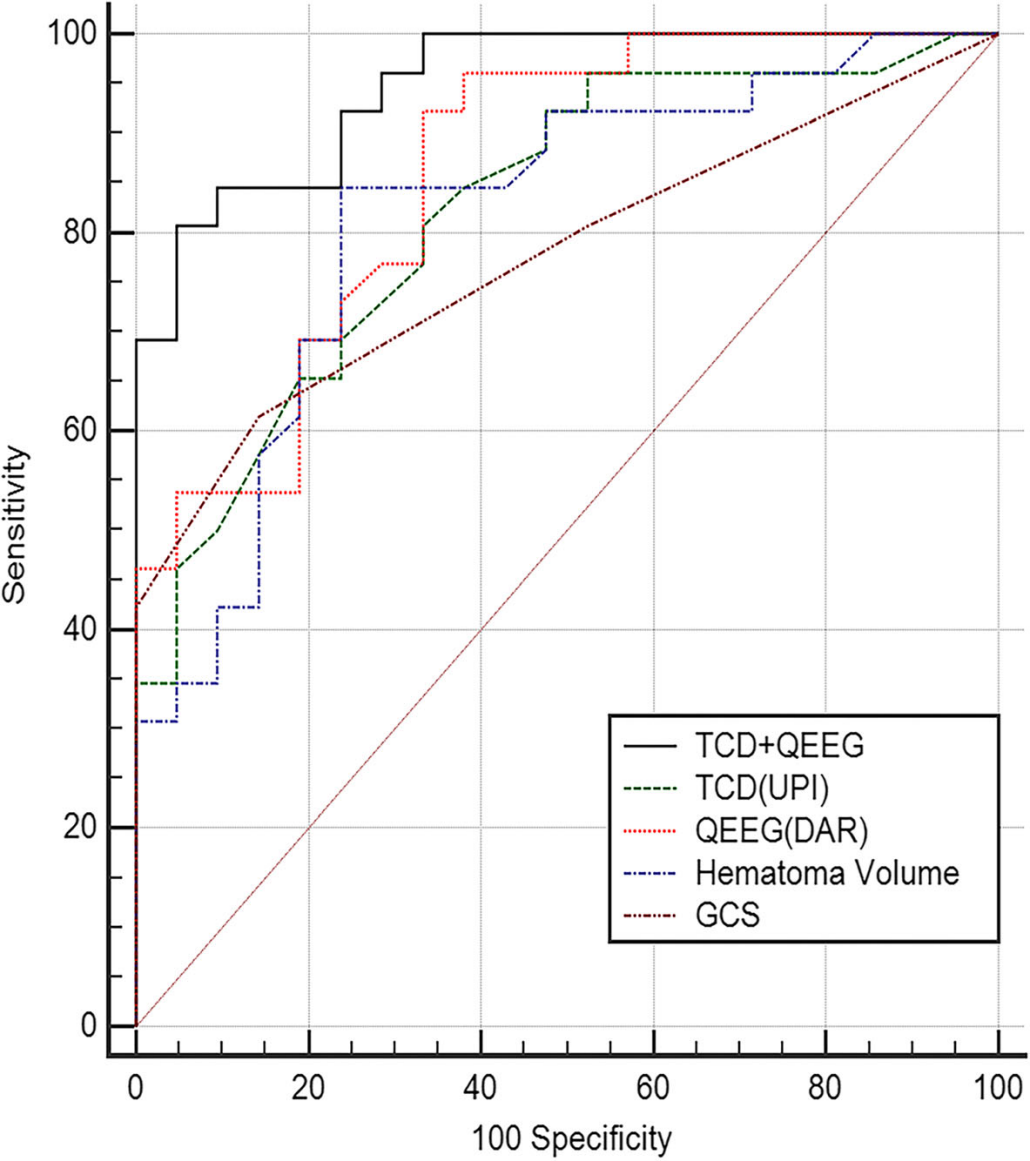
Comparison of ROC curves to predict outcome in this cohort between five models: Glasgow Coma Scale (GCS), AUROC 0.776 (0.630–0.884); hematoma volume, AUROC 0.816 (0.676–0.914); unaffected side pulsatility index (UPI), AUROC 0.822 (0.683–0.918); delta/alpha ratio (DAR), AUROC 0.860 (0.728–0.944); transcranial Doppler (TCD) + quantitative electroencephalography (QEEG), AUROC 0.949 (0.842–0.992). *p* < 0.05 for TCD (UPI) + QEEG (DAR) comparison with GCS, hematoma volume, UPI (independent predictor of TCD), and DAR (independent predictor of QEEG). AUROC area under the receiver operating curve

To determine whether the combination of TCD and QEEG variations in the model improved outcome prediction, we compared the ROC curves of five models: the first model was obtained by the GCS score, the second model contained the hematoma volume, the third model was obtained by the independent predictors of TCD with UPI, the fourth model was obtained by the independent predictors of QEEG with DAR, and the final model included both UPI and DAR. All models could predict mortality in patients with ICH at 90 days. The final model attained a high prognostic power, as the area under the ROC curve was 0.949 when UPI and DAR were combined. The contribution of the final model was significant, while each variable alone did not seem to be significant (Fig. 3).

## Discussion

To our knowledge, this is the first study that focused on combined application of TCD-QEEG to assess the brain function of patients with SAS-ICH, and its prospective design could provide 90-day prognostic information. The results mainly suggested that TCD parameters of response to ICP and QEEG parameters of response to CBF and brain metabolism were significantly changed; UPI in TCD and DAR in QEEG were two independent predictors for 90-day mortality. Moreover, the area under the ROC curve after the combination of UPI and DAR was 0.949, which was superior to any single variable.

In previous studies, a variety of scales based on clinical data have often been used to assess ICH mortality and prognosis; variables that enter the scale usually include age, NIHSS score, GCS score, blood glucose, location of ICH, hematoma volume, intraventricular hemorrhage, and others [21–23]. After analyzing these clinical variables, we found that only GCS score and hematoma volume were associated with mortality. However, after entering these factors into the multiple regression model, they were not independent predictors. The reason may be that we enrolled patients with coma with GCS score ≤ 8, where GCS score and hematoma volume no longer have significant advantage in predicting prognosis in severe ICH.

TCD can assess intracranial compliance and, to some extent, reflect the level of ICP when it increases. The reason for the increase in ICP may be through a rise in intracranial volume, or secondarily through acute obstructive hydrocephalus [24]. The progressive increase in ICP and the decrease in cerebral perfusion pressure (CPP) significantly affect the Doppler waveform; typical changes include decreased Vd and increased PI. Mayer et al.’s [5] study showed that alterations in PI more reliably reflected intracranial lesion volume, and TCD could assess the asymmetry of intracranial hemodynamics. Martí-Fàbregas et al. [6] suggested that TCD is effective for assessing intracranial hypertension, and elevated UPI can predict 30-day outcome in patients with ICH. Kiphuth et al. [7] found that early PI monitoring by TCD correlated with ICP, and may be used to predict the outcome after 6 months. Our study on TCD drew a similar conclusion; decreased UVd and increased bilateral PI were significantly correlated with 90-day mortality, and API and UPI were the more significant predictors. Multivariate regression analysis showed that UPI was an independent prognostic factor.

QEEG is capable of quantitatively reflecting changes in intracranial neuronal activity, CBF, and metabolism. Powers used PET-measured CBF and CMRO_2_ in patients with ICH, and found that both perihematomal CBF and CMRO_2_ were significantly reduced compared to the contralateral side [25]. QEEG changes after ICH have not been reported; many QEEG studies of ischemic stroke have confirmed that QEEG correlates well with CBF and brain metabolism (CMRO_2_) in early subacute ischemic stroke. Finnigan et al. [26] assessed patients with acute supratentorial infarction, and suggested that DAR and RAP were positively correlated with the 30-day NIHSS score. Cuspineda et al. [27] reported (sub)acute ischemic stroke RDP to be the most significant and RAP the next best predictor of the 3-month outcome. Finnigan et al. [28] found that DAR showed maximal accuracy for discriminating between patients with acute ischemic stroke and controls. Our study also showed that the slower frequency band delta power increased and the faster frequency band alpha power decreased with the aggravation of brain injury. RDP, RAP, DAR, and DTABR were all significantly correlated with 90-day mortality; DAR and DTABR were the most significant among them. Multivariate regression analysis showed that DAR was an independent prognostic factor.

Many questions remain regarding the pathophysiological changes in ICH. Severely impaired consciousness is more common with acute supratentorial ICH than with supratentorial infarction ischemic stroke [8]. Alterations in consciousness following ICH are not always easily explained by increased ICP or herniation. We also found that in some patients with severe supratentorial ICH, clinical indications, state of consciousness, and QEEG indicate that the patient is in a state of exhaustion, but the TCD waveform is normal, and PI is not high in the last few hours of monitoring. Additionally, we found that there was no difference in BSI between severe ICH and healthy controls in QEEG. The BSI is an indicator of bilateral hemisphere damage symmetry, while in ischemic stroke the BSI or pairwise-derived BSI significantly increased, and the degree of increase was significantly related to prognosis [29, 30]. It may, therefore, be assumed that brain damage in hemorrhagic stroke is diffuse, unlike in ischemic stroke. Neuroimaging has provided objective evidence of damage to the contralateral hemisphere in unilateral ICH. Zazulia et al. [31] reported swelling of the bilateral hemispheres during the first week following acute supratentorial ICH.

Our study aimed at evaluating the brain function of severe supratentorial ICH using TCD combined with QEEG, and obtained good results, which can be utilized in future research in numerous ways. First, we hope to expand the sample size and obtain the boundary value, which could better guide clinical applications. Second, we could compare brain function of TCD-QEEG with functional imaging, and intensively study the mechanism of ICH. Third, a series of studies have confirmed that there are many similarities between acute ICH and TBI in pathogenesis and clinical manifestations. If TCD and QEEG monitoring of these two groups of patients are performed simultaneously, perhaps we can provide a new basis for the disease pathogenesis. Finally, with the undergoing intensive studies on the pathophysiological mechanisms that follow ICH, new treatment methods are bound to appear. TCD combined with QEEG can dynamically, rapidly, and in a timely manner respond to the changes in brain function before and after treatment.

This study has some limitations. First, this was a single-center study in a university hospital setting, and the sample was relatively small. Thus, prospective validation in different settings and with larger samples is needed. Second, we only monitored the patients in the acute phase, and did not perform dynamic monitoring, which is helpful in comprehensively understanding the changes of brain function with disease progression. Well-designed multicenter TCD combined with QEEG dynamic monitoring studies with a larger sample are urgently needed in the future to provide more information for understanding brain functional changes after ICH.

## Conclusions

This study demonstrated that in patients with SAS-ICH, brain damage caused diffuse changes, evident with multimodality neuromonitoring of TCD combined with QEEG; UPI and DAR were independent predictors of 90-day clinical outcome. Diagnostic power after combined use of TCD-QEEG is statistically superior in performance to any single variable, whether clinical or neurophysiological.

## Abbreviations

APTT: Activated partial thromboplastin time
BSI: Brain symmetry index
CBF: Cerebral blood flow
DAR: Delta/alpha ratio
DTABR: (Delta + theta)/(alpha + beta)ratio
GCS: Glasgow Coma Scale
ICH: Intracerebral hemorrhage
ICP: Intracranial pressure
INR: International Normalized Ratio
PI: Pulsatility index
QEEG: Quantitative electroencephalography
RAP: Relative alpha power
RBP: Relative beta power
RDP: Relative delta power
RTP: Relative theta power
TCD: Transcranial Doppler
Vd: Diastolic flow velocity
Vm: Mean flow velocity
Vs: Systolic flow velocity
